# Efficacy analysis of empirical bismuth quadruple therapy, high-dose dual therapy, and resistance gene-based triple therapy as a first-line *Helicobacter pylori* eradication regimen – An open-label, randomized trial

**DOI:** 10.1515/med-2023-0722

**Published:** 2023-07-11

**Authors:** Xin Jiang, Bin Deng, Xuefeng Gao, Yun Zhang, Guangyao Li, Guiqing Li, Qiang She, Yanbing Ding

**Affiliations:** Department of Gastroenterology, The Affiliated Hospital of Yangzhou University, Yangzhou University, Yangzhou, China; Institute of Gastroenterology, Affiliated Hospital of Yangzhou University, Yangzhou University, Jiangsu, China; Institute of Gastroenterology, Affiliated Hospital of Yangzhou University, Yangzhou University, Yangzhou, China; Department of Emergency Medicine, Suqian Hospital of Nanjing Drum Tower Hospital Group, Suqian, China

**Keywords:** *Helicobacter pylori*, empirical bismuth quadruple therapy, high-dose dual therapy, resistance-based triple therapy, eradication efficacy

## Abstract

This research aimed to evaluate the eradication efficacy, safety, and gastrointestinal symptom relief rates of empirical bismuth quadruple therapy, high-dose dual therapy, and resistance gene-based triple therapy in primary eradication patients in Yangzhou, China. It also investigated the possible factors influencing the success of different *H*elicobacter *pylori* eradication regimens. A single-center, prospective, open-label, randomized controlled study was performed from December 2020 and October 2021, in which 255 patients with *H. pylori* infection were assigned in a 1:1:1 ratio to the three different groups. Our results showed that high-dose dual therapy (91.0%, 71/78) and resistance gene-based triple therapy (94.9%, 75/79) achieved eradication rates and compliance equivalent to those of empirical bismuth quadruple therapy (85.3%, 64/75) in the per-protocol analysis, while high-dose dual therapy had lower rates of adverse events (11.5%, 9/78, *P* < 0.05), fewer side effects, and greater safety. Most patients’ gastrointestinal discomfort symptoms improved after eradication of *H. pylori*. Poor compliance (*P* < 0.05) and antibiotic resistance (*P* < 0.05) were risk factors for the efficacy of *H. pylori* eradication. Therefore, the appropriate regimen can be individualized for eradication therapy in clinical practice according to the patient’s resistance and tolerance to the drug.

## 1 Introduction

*Helicobacter pylori* is a gram-negative bacillus with a high infection rate and high pathogenicity. The prevalence of *H. pylori* infection in the Chinese population is nearly 50% [1], while it has slightly decreased in recent years, the rate is still high [2]. With the widespread use of antibiotics, the rate of antibiotic resistance in our population is also increasing [3]. At present, in the Chinese population, clarithromycin (resistance rate 20–40%), metronidazole (resistance rate 40–70%), and levofloxacin (resistance rate 20–40%) have high resistance rates, while the resistance rates of amoxicillin (0–5%), tetracycline (0–5%), and furazolidone (0–3%) are still low [4]. The eradication rate of standard triple therapy has been reduced to unacceptable levels (eradication rate <80%) as the therapy has been widely abandoned [5]. In the Maastricht V consensus report, it was proposed [6] that classical bismuth quadruple therapy is recommended as first-line eradication therapy in areas with high clarithromycin resistance (>15%). Since tetracycline is difficult to obtain in most parts of mainland China classical bismuth quadruple therapy cannot be widely used. Bismuth-containing quadruple therapy is recommended in the fifth national expert consensus on *H. pylori* infection in China [7], of which the most commonly used regimen with relatively fewer adverse reactions includes proton pump inhibitors (PPI) + amoxicillin + clarithromycin + bismuth. However, in our previous epidemiological survey, the resistance rate to clarithromycin reached 43.65% in the areas of Yangzhou, China. If such therapy was used, the eradication rate of *H. pylori* would inevitably be reduced and nearly half of the patients misused the antibiotics. The efficacy of clarithromycin-containing bismuth quadruple therapy has not been reported in adults in the areas of Yangzhou, China. In addition, there are many types of quadruple therapy drugs and patients have greater concerns about the occurrence of adverse drug events, which greatly affect medication compliance. Thus, we need to explore *H. pylori* eradication options that are more appropriate for the patients in the areas of Yangzhou, China.

When we analyzed the influential factors associated with *H. pylori* eradication failure, we found that antibiotic resistance was one of the most important causes of eradication failure [8]. As an infectious disease, *H. pylori* should theoretically be eradicated by selecting sensitive antibiotics based on susceptibility testing or local antibiotic resistance profiles. A study from Korea [9] aimed to evaluate the efficacy of drug susceptibility-based individualized therapy for first-line eradication of *H. pylori* in regions with high antibiotic resistance, with an intention-to-treat (ITT) eradication rate of 93.1% and a per-protocol (PP) eradication rate of 100.0%. Compared to sequential therapy, the eradication rate of drug susceptibility-based individualized therapy in the population was significantly higher. However, the difference in the antibiotic resistance spectrum in various regions, combined with the strict conditions of *H. pylori* culture and the low success rate of the sensitivity test, limits the selection of medication for *H. pylori* based on drug sensitivity tests [10]. With the rapid development of molecular biology techniques, antibiotic resistance in *H. pylori* can be obtained by genetic testing [11]. A multicenter prospective randomized controlled trial including 526 patients [12] was used to detect *H. pylori* infection and clarithromycin and levofloxacin resistance by a molecular detection method of genotype HelicoDR test, and sensitive antibiotic eradication was selected according to its drug resistance results. The individualized eradication efficiency was higher than that of traditional triple therapy. This molecular biological detection method greatly solves the bottleneck associated with the limited use of drug sensitivity tests in clinical practice.

In our population, the rate of resistance to amoxicillin is very low, and amoxicillin is widely used in infectious diseases [13]. Through an in-depth study of the bactericidal mechanism of amoxicillin, it was found that the antibacterial effect of amoxicillin on *H. pylori* is pH-dependent [14]. When gastric acid is fully inhibited and the pH level in the stomach can continue to reach greater than 6, amoxicillin can fully exploit its bactericidal effect on *H. pylori*; more sufficient gastric acid secretion inhibition yields a greater antibacterial efficacy of amoxicillin. A randomized controlled study [15] from a large sample compared the eradication safety of high-dose dual therapy with that of bismuth quadruple therapy. Modified dual therapy at a high dose and high administration frequency was equally effective, safer, and less costly than bismuth-containing quadruple therapy.

Currently, there is a lack of data regarding the appropriate *H. pylori* eradication regimen in the areas of Yangzhou, China. The aim of this study is to determine whether high-dose dual therapy and resistance-based triple therapy are superior to empirical bismuth quadruple therapy in terms of eradication rates and incidence of adverse events, and to assess the factors that may affect the success of eradication.

## 2 Materials and methods

### 2.1 Study population

The study participants included those who took part in the screening program for upper gastrointestinal tumors in Yangshou Town, Hanjiang District, Yangzhou City between December 2020 and October 2021. All patients underwent *H. pylori* detection and gastroscopy. Patients were considered eligible for enrollment if they were 18–70 years old and had *H. pylori* infection. *H. pylori* infection was defined as both positive for *H. pylori* by ^14^C-UBT and successful detection of the *H. pylori UreA* gene. Patients with any one of the following criteria were excluded: (1) *H. pylori* negative by ^14^C-UBT, or *H. pylori UreA* was not detected; (2) previously received *H. pylori* eradication therapy; (3) had taken H_2_ receptor blockers, PPI, bismuth, antibiotics, or other drugs with antibacterial effects in the past 4 weeks; (4) allergies or have drug contraindications for PPI, bismuth, amoxicillin, clarithromycin, metronidazole, levofloxacin, furazolidone, or tetracycline drugs used in this study; (5) pregnant or lactating women; (6) history of malignant tumors or gastric surgery; (7) serious liver disease, kidney disease, cardiovascular and cerebrovascular diseases, lung disease, blood diseases, or other serious diseases affecting the evaluation of this study; and (8) unwilling or unable to sign the consent form.

This study protocol was approved by the Ethics Committee of the Affiliated Hospital of Yangzhou University (2020-YKL11-10) and was conducted in accordance with the principles of the Declaration of Helsinki and the standards of Good Clinical Practice. Informed consent was obtained from each participant. This study was registered in the China Clinical Trial Registry (ChiCTR2000040469).

### 2.2 Study design

This was a single-center, prospective, open-label, randomized controlled study. According to the order of presentation, patients who met the inclusion criteria were divided into an empirical bismuth quadruple therapy group, a high-dose dual therapy group, and a resistance gene-based triple therapy group at a ratio of 1:1:1 using a random number table. We collected general condition data, clinical data, and gastric mucosal tissue of the patients included in the study and performed gastroscopy, ^14^C-UBT, and *H. pylori* resistance gene detection. This was an open-label study in which both investigators and patients were aware of the eradication regimen; staff performing epidemiological investigations, gastroscopy, ^14^C-UBT, and *H. pylori* resistance gene testing were blinded to group assignment to avoid possible measurement bias.

All patients returned within 1 week after the end of the radical course of treatment, and the patients were asked about the adverse events during treatment to determine the incidence of these adverse events. The remaining drugs in the patients’ bodies were examined to assess medication compliance. After discontinuation for at least 1 month, all patients underwent a repeat ^14^C-UBT to assess *H. pylori* infection status and to determine whether eradication of *H. pylori* was successful.

### 2.3 Treatment regimens

**Figure 1:**
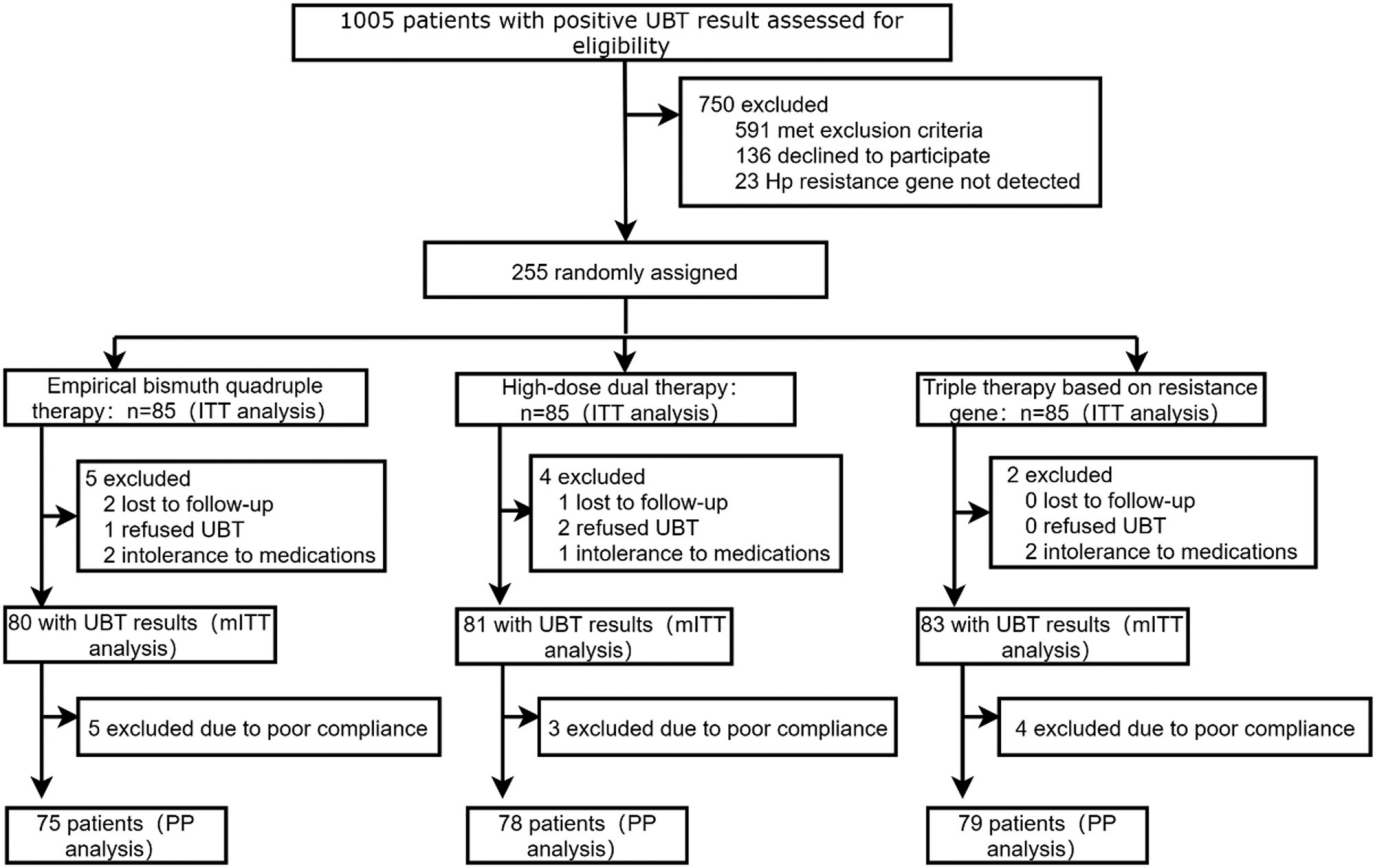
Study flowchart. ITT, intention-to-treat; mITT, modified intention-to-treat; PP, per-protocol.

**Figure 2:**
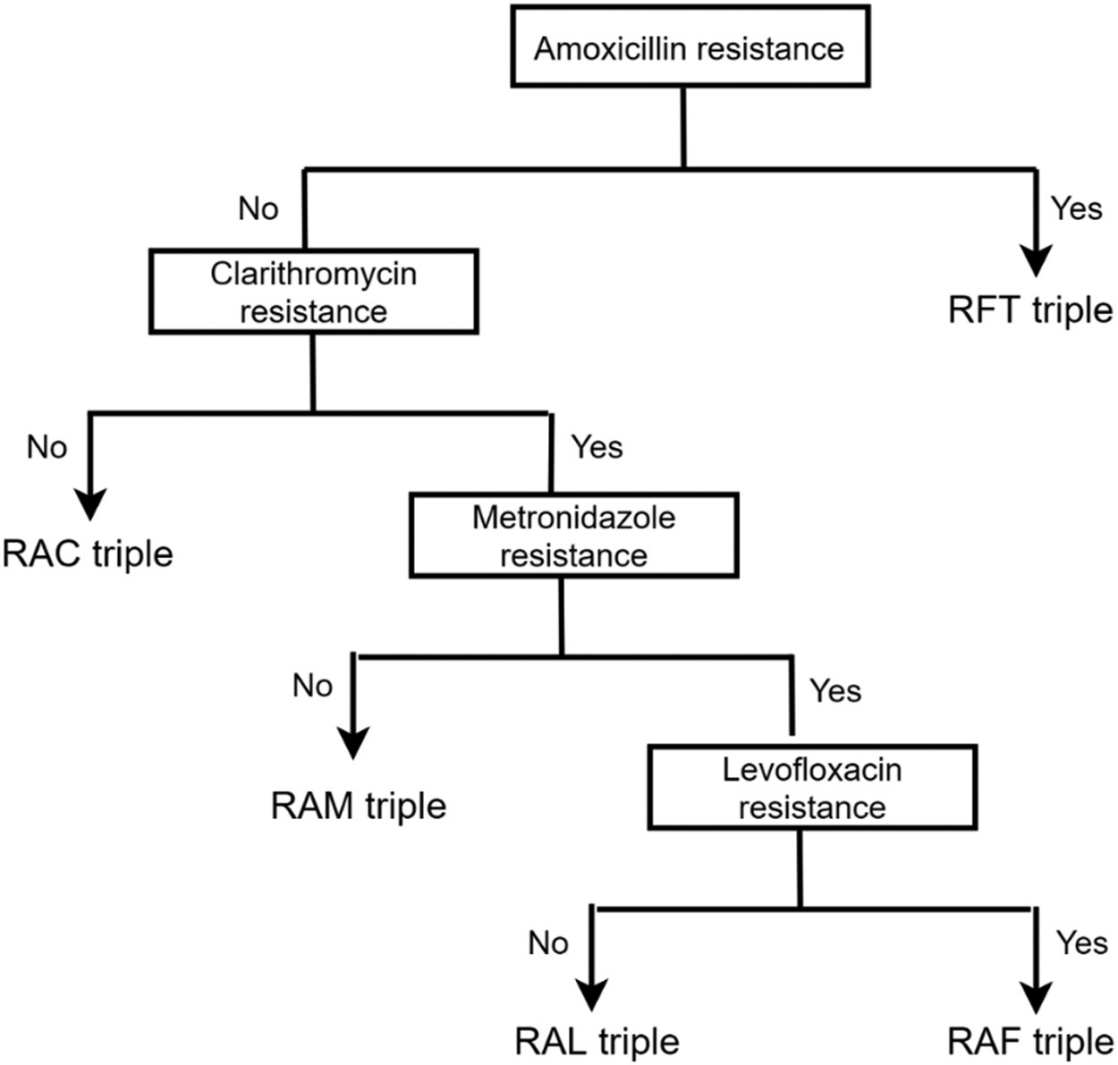
Procedures for selecting sensitive antibiotic therapy based on *H. pylori* resistance gene test results. R, rabeprazole; A, amoxicillin; C, clarithromycin; M: metronidazole; L: levofloxacin; F: furazolidone; T, tetracycline.

The groups and intervention strategies of this study are shown in Figures 1 and 2, and patients who met the inclusion criteria were assigned to empirical bismuth quadruple therapy, high-dose dual therapy, and resistance gene-based triple therapy groups. Radical therapy for patients in the empirical bismuth quadruple therapy group (RACB regimen) was as follows: rabeprazole 20 mg, colloidal bismuth pectin 220 mg, amoxicillin 1,000 mg, and clarithromycin 500 mg all twice daily. Radical therapy for patients in the high-dose dual therapy group (RA regimen) was as follows: rabeprazole 20 mg and amoxicillin 750 mg four times daily. Lastly, radical therapy for patients in resistance gene-based triple therapy was as follows: if the patient was resistant to amoxicillin, rabeprazole 20 mg twice daily, furazolidone 100 mg twice daily, and tetracycline 500 mg three times daily (RFT regimen); if the patient was sensitive to amoxicillin and clarithromycin, rabeprazole 20 mg, amoxicillin 1,000 mg, and clarithromycin 500 mg all twice daily (RAC regimen); if the patient was sensitive to amoxicillin and metronidazole, but was resistant to clarithromycin, rabeprazole 20 mg twice daily, amoxicillin 1,000 mg twice daily, and metronidazole 400 mg three times daily (RAM regimen); if the patient was sensitive to amoxicillin and levofloxacin, but was resistant to clarithromycin and metronidazole, rabeprazole 20 mg twice daily, amoxicillin 1,000 mg twice daily, and levofloxacin 500 mg once daily (RAL regimen); and if the patient was sensitive to amoxicillin, but was resistant to clarithromycin, metronidazole, and levofloxacin, rabeprazole 20 mg, amoxicillin 1,000 mg, and furazolidone 100 mg, all twice daily (RAF regimen).

The course of radical treatment in all three groups was 14 days, during which rabeprazole and colloidal bismuth pectin were administered half an hour before meals; amoxicillin, clarithromycin, metronidazole, levofloxacin, furazolidone, and tetracycline were administered half an hour after meals; and the drugs that needed to be taken four times daily were taken at bedtime in addition to the three meal times.

### 2.4 *H. pylori* detection and resistance gene detection

All eligible participants underwent an upper endoscopy and ^14^C-UBT after entering the study. A gastric biopsy taken from the antrum was subjected to *H. pylori UreA, vacA* gene detection as well as resistance gene detection (Hangzhou Meilian Medical Laboratory Co., Ltd). Antibiotics targeted by the resistance genes included amoxicillin (PBP1), clarithromycin (23S rRNA), metronidazole (rdxA), and levofloxacin (gyrA).

^14^C-UBT was used to confirm the presence of *H. pylori* infection at least 4 weeks after treatment. The participants swallowed one ^14^C-urea capsule in a fasting state and sat quietly for 20 min. During this period, participants did not exercise, eat, or drink. After this period, the participants were instructed to gently blow air into a vial with a red indicator through a disposable plastic expiratory tube for 1–3 min. The red indicator disappeared and the vial cap was closed. Scintillation fluid was added first during the measurement, and the disintegrations per minute (DPM) value of the participant was measured for 3 min on the *H. pylori* tester to determine whether the participant was infected with *H. pylori*. If the DPM was ≥100, the patient was considered positive for *H. pylori*.

### 2.5 Safety and compliance

All participants were informed of medication-related matters, including drug type, dose, frequency, medication time, and possible adverse events, in-person before medication administration. Each participant was given a record sheet of adverse events during medication administration. The participants recorded their medication and adverse events every day. The investigator could be contacted by telephone at any time if the participant had obvious discomfort during medication. It was recommended that the drug should not be discontinued in cases of mild-to-moderate adverse events. We asked the participants to return 1–3 days after eradication for compliance assessment and to determine the incidence of adverse effects. After taking the drug on time for 2 weeks and stopping all drugs for at least 4 weeks, we instructed the participants to perform ^14^C-UBT to determine whether the eradication of *H. pylori* was successful. The definition of compliance was ≥80% of all study drugs taken as instructed; otherwise, the patient was considered to have poor compliance.

### 2.6 Statistical analysis

The main aim of this study was to compare empirical bismuth quadruple therapy with a control. During the pre-test, 20 patients in each group underwent *H. pylori* eradication, and the eradication rates were 75, 86, and 93% in the empirical bismuth quadruple therapy, high-dose dual therapy group, and resistance gene-based triple therapy, respectively. According to the purpose and design of this study, the hypothesis test type I error *α* was 0.05, type II error *β* was 0.2, and the sample size ratio of each group was 1:1:1, assuming the failure rate was 10–15%. It was determined that 85 patients were required in each group for the three radical treatment regimens.

All participants were included in the ITT analysis. The participants who had taken the drug at least once and had the reexamination result from the ^14^C-UBT were included in the mITT analysis. The participants with good compliance and reexamination results from the ^14^C-UBT were included in the PP analysis.

Statistical analysis was performed using SPSS for Windows (version 21.0; IBM, Armonk, NY, USA). Two-sided *P* < 0.05 was considered to be statistically significant when comparing the three groups. Adjustment for multiple comparisons was made by setting a Bonferroni-corrected *P* level of 0.017. Categorical variables were described as percentages or frequencies, while continuous variables were described as mean ± standard deviation. The eradication rates and 95% CI were calculated. Intergroup differences were evaluated using Pearson chi-square or Fisher’s exact test for categorical variables and Student’s *t* test for continuous variables. Univariate analysis was performed to evaluate significant predictive variables for *H. pylori* eradication in participants with mITT analysis. A multiple logistic regression analysis was performed using variables with statistical significance in univariate analysis. Odds ratios (OR) and 95% CI for unsuccessful eradication were calculated according to the different variables.

## 3 Results

### 3.1 Characteristics of the participants

According to the inclusion and exclusion criteria, 255 participants were enrolled in this study from December 2020 to October 2021, and were assigned in a 1:1:1 ratio to the empirical bismuth quadruple therapy, high-dose dual therapy, and resistance gene-based triple therapy groups, with 85 patients in each group. Among the empirical bismuth quadruple therapy, two patients were lost to follow-up, one patient refused to have reexamination of ^14^C-UBT, two patients were intolerant to adverse drug reactions and discontinued halfway, and finally, 80 patients were included in the mITT analysis; excluding five patients with poor compliance, a total of 75 patients were included in the PP analysis. Among the high-dose dual therapy, one patient was lost to follow-up, two patients refused to have reexamination of ^14^C-UBT, one patient was intolerant to adverse drug reactions and discontinued halfway, and finally, 81 patients were included in the mITT analysis; excluding three patients with poor compliance, a total of 78 patients were included in the PP analysis. Among the resistance gene-based triple therapy, two patients were intolerant to adverse drug reactions and discontinued halfway, and finally, 83 patients were included in the mITT analysis; excluding four patients with poor compliance, a total of 79 patients were included in the PP analysis. The baseline characteristics of participants collected in each group included: sex, age, body mass index, family population, systolic blood pressure, diastolic blood pressure, smoking history, drinking history, hypertension, diabetes, hyperlipidemia, dietary habits, family history of gastrointestinal cancer, previous history of dyspepsia, endoscopic diagnosis, pathological diagnosis, *H. pylori* virulence gene, antibiotic resistance, and antibiotic resistance pattern. Except for diastolic blood pressure, there were no significant differences in other baseline characteristics among the three groups in the ITT analysis population. Specific baseline characteristics are shown in Table 1.

**Table 1:** Comparison of baseline data between three therapy groups

Baseline factors	Empirical bismuth quadruple therapy (*n* = 85)	High-dose dual therapy (*n* = 85)	Resistance gene-based triple therapy (*n* = 85)	*P* value
Sex, *n*/*N* (%)				0.119
Male	44/85(51.8%)	31/85(36.5%)	35/85(41.2%)	
Female	41/85(48.2%)	54/85(63.5%)	50/85(58.8%)	
Age, mean ± SD, years	54.42 ± 8.85	53.61 ± 8.59	53.51 ± 8.20	0.746
BMI, mean ± SD, kg/m^2^	23.79 ± 2.65	24.49 ± 2.93	24.38 ± 3.08	0.239
Family populations, *n*/*N* (%)				0.182
≤3	45/85(52.9%)	33/85(38.8%)	39/85(45.9%)	
>3	40/85(47.1%)	52/85(61.2%)	46/85(54.1%)	
Systolic blood pressure, mean ± SD, mmHg	131.22 ± 14.62	134.48 ± 11.81	131.13 ± 13.75	0.180
Diastolic blood pressure, mean ± SD, mmHg	84.21 ± 7.23	87.06 ± 6.76	84.48 ± 7.74	0.020*
Smoking, *n*/*N* (%)	28/85(32.9%)	19/85(22.4%)	30/85(35.3%)	0.147
Alcohol intake, *n*/*N* (%)	34/85(40.0%)	30/85(35.3%)	25/85(29.4%)	0.349
Hypertension, *n*/*N* (%)	20/85(23.5%)	26/85(30.6%)	21/85(24.7%)	0.534
Diabetes, *n*/*N* (%)	3/85(3.5%)	5/85(5.9%)	3/85(3.5%)	0.796
Hyperlipemia, *n*/*N* (%)	7/85(8.2%)	8/85(9.4%)	2/85(2.4%)	0.142
Dietary habits, *n*/*N* (%)				0.467
Regular diet	76/85(89.4%)	73/85(85.9%)	78/85(91.8%)	
Irregular diet	9/85(10.6%)	12/85(14.1%)	7/85(8.2%)	
Feeding rate, *n*/*N* (%)				0.339
Normal	66/85(77.6%)	69/85(81.2%)	61/85(71.8%)	
Too fast or too slow	19/85(22.4%)	16/85(18.8%)	24/85(28.2%)	
Food temperature, *n*/*N* (%)				0.716
Moderate	76/85(89.4%)	77/85(90.6%)	79/85(92.9%)	
Too hot or too cold	9/85(10.6%)	8/85(9.4%)	6/85(7.1%)	
Family history of gastrointestinal cancer, *n*/*N* (%)				0.269
Yes	28/85(32.9%)	31/85(36.5%)	38/85(44.7%)	
No	57/85(67.1%)	54/85(63.5%)	47/85(55.3%)	
Prior history of dyspepsia, *n*/*N* (%)				0.606
Yes	26/85(30.6%)	21/85(24.7%)	21/85(24.7%)	
No	59/85(69.4%)	64/85(75.3%)	64/85(75.3%)	
Endoscopic diagnosis, *n*/*N* (%)				0.336
Peptic ulcer	13/85(15.3%)	7/85(8.2%)	9/85(10.6%)	
Nonpeptic ulcer	72/85(84.7%)	78/85(91.8%)	76/85(89.4%)	
Pathological diagnosis, *n*/*N* (%)				0.212
Chronic superficial gastritis	22/85(25.9%)	28/85(32.9%)	19/85(22.4%)	
Chronic atrophic gastritis	43/85(50.6%)	45/85(52.9%)	40/85(47.1%)	
Intestinal metaplasia	17/85(20.0%)	11/85(12.9%)	20/85(23.5%)	
Atypical hyperplasia	3/85(3.5%)	1/85(1.2%)	6/85(7.1%)	
*Vac A*, *n*/*N* (%)				0.539
s1/m1	32/85(37.6%)	32/85(37.6%)	26/85(30.6%)	
s1/m2	53/85(62.4%)	53/85(62.4%)	59/85(69.4%)	
Antibiotic resistance, *n*/*N* (%)				
Amoxicillin resistance	8/85(9.4%)	7/85(8.2%)	5/85(5.9%)	0.684
Clarithromycin resistance	33/85(38.8%)	42/85(49.4%)	48/85(56.5%)	0.068
Levofloxacin resistance	44/85(51.8%)	32/85(37.6%)	45/85(52.9%)	0.063
Metronidazole resistance	29/85(34.1%)	32/85(37.6%)	37/85(43.5%)	0.444
Antibiotic resistance patterns, *n*/*N* (%)				
All sensitive	20/85(23.5%)	21/85(24.7%)	12/85(14.1%)	0.176
Single resistance	27/85(31.8%)	21/85(24.7%)	26/85(30.6%)	0.554
Double resistance	27/85(31.8%)	37/85(43.5%)	33/85(38.8%)	0.282
Multidrug resistance	11/85(12.9%)	6/85(7.1%)	14/85(16.5%)	0.165

SD, standard deviation; BMI, body mass index; *P < 0.05.

### 3.2 Eradication rates

**Figure 3:**
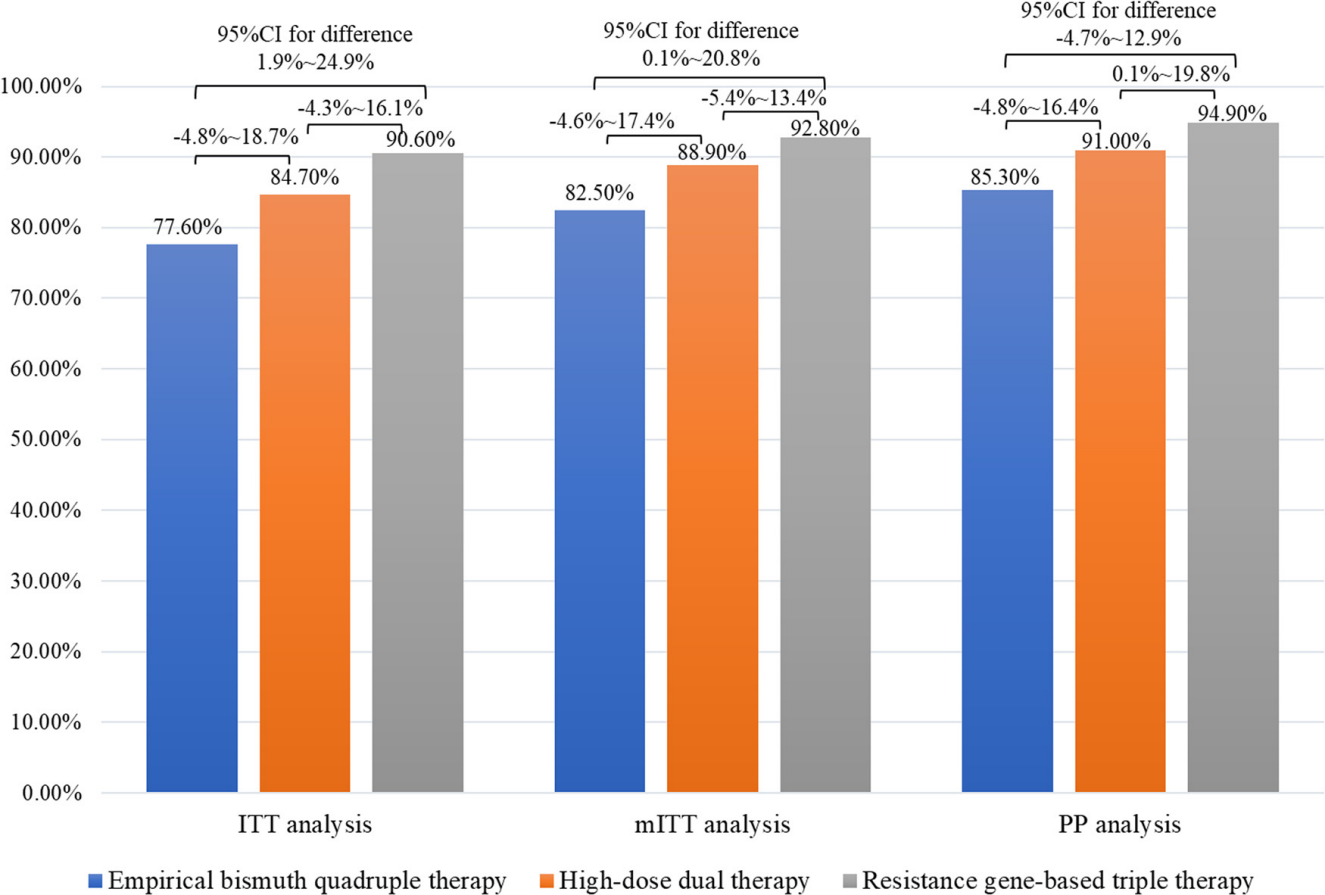
Comparison of eradication rates between three therapy groups.

The primary outcome indicator of this study was to determine whether high-dose dual therapy and resistance gene-based triple therapy were superior to empirical bismuth quadruple therapy for *H. pylori* eradication. The eradication rate of resistance gene-based triple therapy was superior to that of empirical bismuth quadruple therapy (90.6 vs 77.6%, respectively, with a rate difference of 13.0% in ITT analysis; 94.9 vs 85.3%, respectively, with a rate difference of 9.6% in PP analysis) (Table 2), but the difference was not statistically significant both in the ITT analysis and PP analysis (Figure 3). Moreover, the eradication rate of the high-dose dual therapy group was superior to that of the empirical bismuth quadruple therapy group (84.7 vs 77.6%, respectively, with a rate difference of 7.1% in the ITT analysis; 91.0 vs 85.3%, respectively, with a rate difference of 5.7% in the PP analysis) (Table 2), and also the difference was not statistically significant (Figure 3). We concluded that high-dose dual therapy and resistance gene-based triple therapy could achieve eradication rates equivalent to the efficacy of empirical bismuth quadruple therapy.

**Table 2:** Comparison of eradication rates between three therapy groups

Eradication rate	Empirical bismuth quadruple therapy		High-dose dual therapy		Resistance gene-based triple therapy		*P* value
	*n*/*N*	95% CI (%)	*n*/*N*	95% CI (%)	*n*/*N*	95% CI (%)	
ITT analysis	66/85(77.6%)	67.7–85.2	72/85(84.7%)	75.6–90.8	77/85(90.6%)	82.5–95.2	0.067
mITT analysis	66/80(82.5%)	72.7–89.3	72/81(88.9%)	80.2–94.0	77/83(92.8%)	85.1–96.6	0.124
PP analysis	64/75(85.3%)	75.6–91.6	71/78(91.0%)	82.6–95.6	75/79(94.9%)	87.7–98.0	0.124

CI, confidence interval; ITT, intention-to-treat; mITT, modified intention-to-treat; PP, per-protocol.

### 3.3 Compliance and adverse events

The compliance and incidence of adverse events among the groups are presented in Table 3. The compliance in both the high-dose dual therapy group and the resistance gene-based triple therapy group was comparable to that of empirical bismuth quadruple therapy. In terms of safety, in the empirical bismuth quadruple therapy group, two patients withdrew from the drug because of unbearable bitter taste, nausea, and dark stools; in the high-dose dual therapy group, one patient withdrew from the drug because of a rash with unbearable itching after eating seafood while taking the drug. In the resistance gene-based triple therapy group, one patient withdrew from the drug owing to dizziness and nausea after drinking alcohol while taking the drug, and one patient withdrew from the drug because of abdominal pain, diarrhea, and other gastrointestinal events. The other patients experienced mild or moderate adverse events and were not given specific treatment. There was a statistical difference in the incidence of adverse events between the three radical regimens (*P* = 0.009), mainly in symptoms such as bitter taste (*P* = 0.016), darkened stools (*P* < 0.001), and nausea (*P* = 0.039). The incidence of adverse events was significantly lower with the high-dose dual therapy than with the empirical bismuth quadruple therapy (*P* = 0.002) and the incidence of adverse events with resistance gene-based triple therapy was equivalent to empirical bismuth quadruple therapy (*P* = 0.272).

**Table 3:** Comparison of safety and compliance between three therapy groups

Safety and compliance	Empirical bismuth quadruple therapy (*n* = 75)	High-dose dual therapy (*n* = 78)	Resistance gene-based triple therapy (*n* = 79)	*P* value
Patients with adverse events	24(32.0%)^#^	9(11.5%)^#^	19(24.1%)	0.009*
Mild	13(17.3%)	6(7.7%)	12(15.2%)	0.181
Moderate	9(12.0%)	2(2.6%)	5(6.3%)	0.068
Severe	2(2.7%)	1(1.3%)	2(2.5%)	0.871
Bitter taste	14(18.7%)^$^	3(3.8%)^$^	10(12.7%)	0.016*
Dark stools	18(24.0)^&^	—	2(2.5%)^&^	<0.001*
Nausea	4(5.3%)	3(3.8%)	11(13.9%)	0.039
Diarrhea	6(8.0%)	5(6.4%)	6(7.6%)	0.925
Constipation	5(6.7%)	1(1.3%)	4(5.1%)	0.224
Abdominal pain/discomfort	3(4.0%)	2(2.6%)	4(5.1%)	0.774
Asthenia	2(2.7%)	1(1.3%)	2(2.5%)	0.871
Decreased appetite	1(1.3%)	1(1.3%)	3(3.8%)	0.624
Rash	3(4.0%)	5(6.4%)	2(2.5%)	0.478
Headache	2(2.7%)	1(1.3%)	1(1.3%)	0.695
Dizziness	1(1.3%)	1(1.3%)	2(2.5%)	1.000
Good compliance	75/85(88.2%)	78/85(91.8%)	79/85(92.9%)	0.537

^#^ *P* = 0.002 for high-dose dual therapy versus empirical bismuth quadruple therapy; ^$^ *P* = 0.004 for high-dose dual therapy versus empirical bismuth quadruple therapy; ^&^ *P* < 0.017 for resistance gene-based triple therapy versus empirical bismuth quadruple therapy; ^*^ *P* < 0.017.

### 3.4 Improvement of gastrointestinal symptoms after eradication therapy

**Figure 4:**
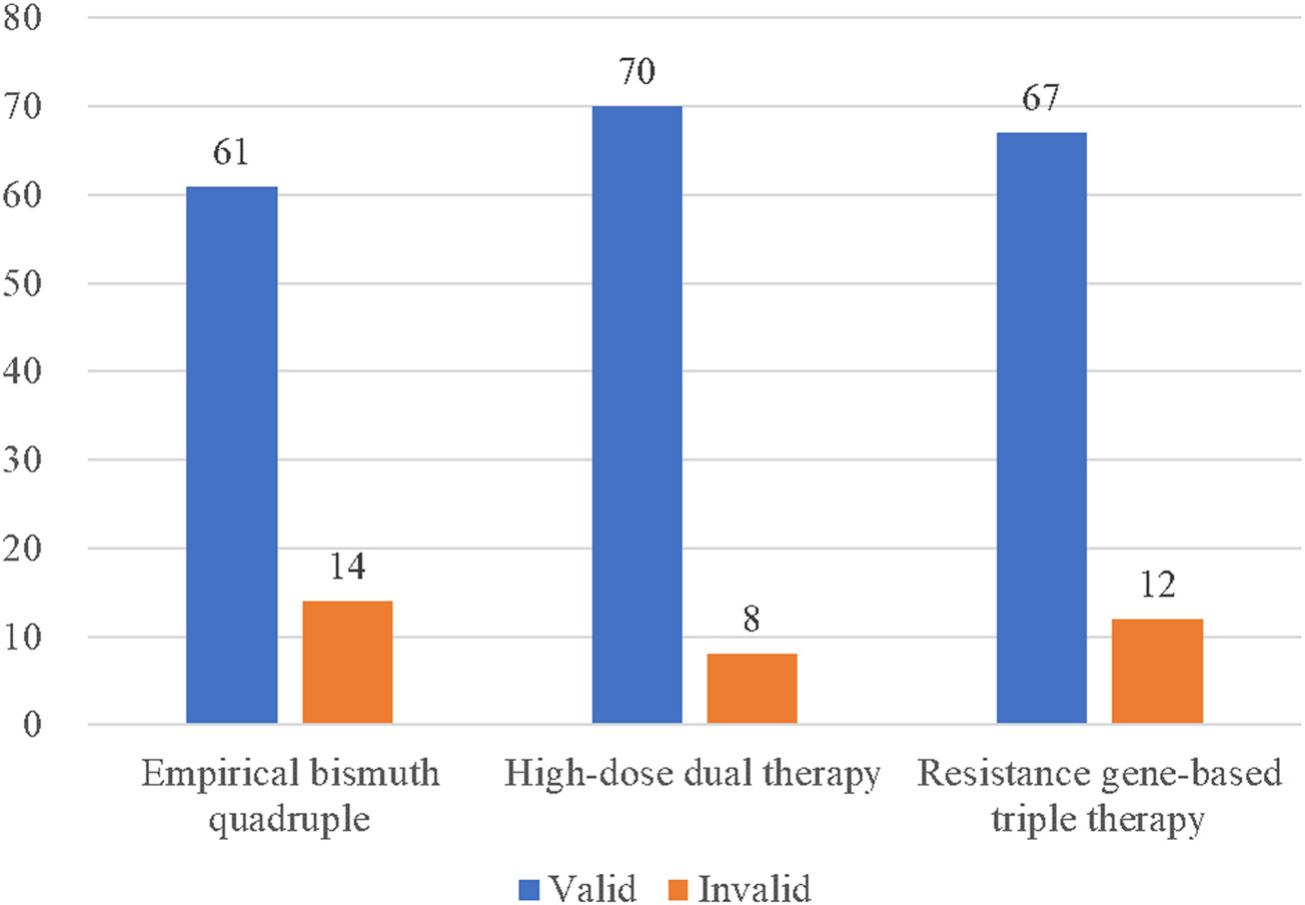
Improvement in gastrointestinal symptoms after *H. pylori* eradication.

The Global Overall Symptom questionnaires were given to participants before the *H. pylori* eradication therapy and after 1 month from the eradication therapy completed. The overall relief of gastrointestinal symptoms after eradication therapy was 81.3% (61/75) in patients treated with the empirical bismuth quadruple therapy, 89.7% (70/78) in patients treated with high-dose dual therapy, and 84.8% (67/79) in patients treated with the resistance gene-based triple therapy (Figure 4). Most patients’ discomfort symptoms improved after eradication of *H. pylori*, and there was no statistical difference between the three groups (*P* > 0.05).

### 3.5 Risk factors for eradication failure

Univariate and multivariate analyses were used to investigate the risk factors for eradication failure. Univariate analysis (Table 4) showed poor compliance (40.0 vs 85.3%, *P* = 0.035), resistance to amoxicillin (50.0 vs 86.1%, *P* = 0.039), resistance to clarithromycin (61.3 vs 95.9%, *P* < 0.001), and multidrug resistance (45.5 vs 88.4%, *P* = 0.002) as risk factors for eradication failure in empirical bismuth quadruple therapy. Meanwhile poor compliance (33.3 vs 91.0%, *P* = 0.031) and resistance to amoxicillin (57.1 vs 91.9%, *P* = 0.027) were shown as risk factors for eradication failure in high-dose dual therapy; and poor compliance (50.0 vs 94.9%, *P* = 0.025) was shown as risk factors for eradication failure in resistance gene-based triple therapy. Multivariate analysis identified poor compliance (OR 13.607, 95% CI 1.249–148.187, *P* = 0.032) and resistance to clarithromycin (OR 12.582, 95% CI 1.925–82.245, *P* = 0.008) as independent predictors of eradication failure in empirical bismuth quadruple therapy. Meanwhile, multivariate analysis identified poor compliance (OR 33.500, 95% CI 2.479–452.762, *P* = 0.008) and resistance to amoxicillin (OR 12.562, 95% CI 2.066–76.391, *P* = 0.006) as independent predictors of eradication failure in high-dose dual therapy. We found no independent predictor of eradication failure in resistance gene-based triple therapy.

**Table 4:** Univariate analysis showing factors affecting *H. pylori* eradication rates

Influencing factors	Empirical bismuth quadruple therapy (*n* = 80)		High-dose dual therapy (*n* = 81)		Resistance gene-based triple therapy (*n* = 83)	
	Eradication rate	*P* value	Eradication rate	*P* value	Eradication rate	*P* value
Gender, *n*/*N* (%)		0.331		0.838		1.000
Male	33/42(78.6%)		25/29(86.2%)		32/34(94.1%)	
Female	33/38(86.8%)		47/52(90.4%)		45/49(91.8%)	
Age, years, *n*/*N* (%)		0.652		0.275		0.483
≤50	20/23(87.0%)		29/33(87.9%)		33/36(91.7%)	
51–60	30/36(83.3%)		27/32(84.4%)		26/29(89.7%)	
>60	16/21(76.2%)		16/16(100.0%)		18/18(100.0%)	
BMI, kg/m^2^, *n*/*N* (%)		0.563		0.268		0.645
≤22	16/19(84.2%)		15/15(100.0%)		13/15(86.7%)	
22–25	31/36(86.1%)		27/30(90.0%)		35/37(94.6%)	
>25	19/25(76.0%)		30/36(83.3%)		29/31(93.5%)	
Family populations, *n*/*N* (%)		0.918		0.157		0.522
≤3	34/41(82.9%)		30/31(96.8%)		34/38(89.5%)	
>3	32/39(82.1%)		42/50(84.0%)		43/45(95.6%)	
Smoking, *n*/*N* (%)		0.221		1.000		1.000
Yes	19/26(73.1%)		15/17(88.2%)		27/29(93.1%)	
No	47/54(87.0%)		57/64(89.1%)		50/54(92.6%)	
Alcohol intake, *n*/*N* (%)		0.400		0.838		0.826
Yes	25/32(78.1%)		25/29(86.2%)		23/24(95.8%)	
No	41/48(85.4%)		47/52(90.4%)		54/59(91.5%)	
Hypertension, *n*/*N* (%)		1.000		0.644		0.986
Yes	16/19(84.2%)		22/26(84.6%)		20/21(95.2%)	
No	50/61(82.0%)		50/55(90.9%)		57/62(91.9%)	
Diabetes, *n*/*N* (%)		0.443		0.454		0.204
Yes	2/3(66.7%)		4/5(80.0%)		2/3(66.7%)	
No	64/77(83.1%)		68/76(89.5%)		75/80(93.8%)	
Hyperlipemia, *n*/*N* (%)		0.539		0.216		1.000
Yes	6/6(100.0%)		6/8(75.0%)		2/2(100.0%)	
No	60/74(81.1%)		66/73(90.4%)		75/81(92.6%)	
Dietary habits, *n*/*N* (%)		1.000		1.000		1.000
Regular diet	59/71(83.1%)		61/69(88.4%)		70/76(92.1%)	
Irregular diet	7/9(77.8%)		11/12(91.7%)		7/7(100.0%)	
Feeding rate, *n*/*N* (%)		1.000		0.879		1.000
Normal	51/62(82.3%)		58/66(87.9%)		56/60(93.3%)	
Too fast or too slow	15/18(83.3%)		14/15(93.3%)		21/23(91.3%)	
Food temperature, *n*/*N* (%)		1.000		1.000		1.000
Moderate	59/71(83.1%)		65/73(89.0%)		71/77(92.2%)	
Too hot or too cold	7/9(77.8%)		7/8(87.5%)		6/6(100.0%)	
Family history of gastrointestinal cancer, *n*/*N* (%)		0.269		1.000		0.834
Yes	20/27(74.1%)		27/30(90.0%)		36/38(94.7%)	
No	46/53(86.8%)		45/51(88.2%)		41/45(91.1%)	
Prior history of dyspepsia, *n*/*N* (%)		0.847		0.893		1.000
Yes	19/24(79.2%)		18/21(85.7%)		19/20(95.0%)	
No	47/56(83.9%)		54/60(90.0%)		58/63(92.1%)	
Endoscopic diagnosis, *n*/*N* (%)		0.621		1.000		0.467
Peptic ulcer	11/12(91.7%)		6/6(100.0%)		7/8(87.5%)	
Nonpeptic ulcer	55/68(80.9%)		66/75(88.0%)		70/75(93.3%)	
Pathological diagnosis, *n*/*N* (%)		0.854		0.352		0.254
Chronic superficial gastritis	18/21(85.7%)		22/26(84.6%)		17/19(89.5%)	
Chronic atrophic gastritis	33/40(82.5%)		41/44(93.2%)		36/39(92.3%)	
Intestinal metaplasia	12/16(75.0%)		8/10(80.0%)		20/20(100.0%)	
Atypical hyperplasia	3/3(100.0%)		1/1(100.0%)		4/5(80.0%)	
Compliance		0.035^*#^		0.031^*$^		0.025^*^
Good	64/75(85.3%)		71/78(91.0%)		75/79(94.9%)	
Poor	2/5(40.0%)		1/3(33.3%)		2/4(50.0%)	
*Vac A*		0.287		0.443		0.443
s1/m1	23/30(76.7%)		26/31(83.9%)		32/33(97.0%)	
s1/m2	43/50(86.0%)		46/50(92.0%)		45/50(90.0%)	
Amoxicillin resistance		0.039^*^		0.027^*$^		
Yes	4/8(50.0%)		4/7(57.1%)		—	
No	62/72(86.1%)		68/74(91.9%)		—	
Clarithromycin resistance		<0.001^*#^				
Yes	19/31(61.3%)		—		—	
No	47/49(95.9%)		—		—	
Levofloxacin resistance		0.118				
Yes	32/42(76.2%)		—		—	
No	34/38(89.5%)		—		—	
Metronidazole resistance		0.511				
Yes	25/29(86.2%)		—		—	
No	41/52(80.4%)		—		—	
All sensitive		0.289				
Yes	16/17(94.1%)		—		—	
No	50/63(79.4%)		—		—	
Single resistance		0.166				
Yes	25/27(92.6%)		—		—	
No	41/53(77.4%)		—		—	
Multidrug resistance		0.002^*^				
Yes	5/11(45.5%)		—		—	
No	61/69(88.4%)		—		—	

BMI, body mass index; ^*^ *P* < 0.05; ^#^Multivariate analysis identified poor compliance (OR 13.607, 95% CI 1.249–148.187, *P* = 0.032) and resistance to clarithromycin (OR 12.582, 95% CI 1.925–82.245, *P* = 0.008) as independent predictors of eradication failure in empirical bismuth quadruple therapy; ^$^Multivariate analysis identified poor compliance (OR 33.500, 95% CI 2.479–452.762, *P* = 0.008) and resistance to amoxicillin (OR 12.562, 95% CI 2.066–76.391, *P* = 0.006) as independent predictors of eradication failure in high-dose dual therapy.

## 4 Discussion

*H. pylori*, an intragastric parasitic bacterium, has highly pathogenic characteristics [16–18]. China has a particularly high burden of *H. pylori* infections; the infection rate varies from region to region, and the prevalence of *H. pylori* infection in adults shows a decreasing trend in urban areas, which seems to be explained by differences in economic and social conditions [19,20]. However, in our previous epidemiological survey, we found that the infection rate of *H. pylori* in the areas of Yangzhou, China, reached 42.36% and belonged to areas with high infection rates. Recent surveillance reports on the trend of antimicrobial resistance in *H. pylori* have confirmed that the incidence of antibiotic resistance is increasing [4,21,22], resulting in a declining eradication rate, and that traditional triple therapy for *H. pylori* eradication is obsolete. Therefore, selection of an appropriate eradication therapy before the initial eradication of *H. pylori* is the focus of clinical attention. The consensus statement recommends [6,23,24] alternative first-line treatment options, including concomitant, sequential, mixed, and bismuth-containing quadruple therapy. Owing to the influence of clarithromycin and metronidazole resistance, non-bismuth quadruple therapy is not applicable in China. Bismuth-containing quadruple therapy is recommended as the first-line eradication regimen in the Fifth National Consensus Report on the Management of *H. pylori* infection in China [7]. It is also the most widely used radical regimen in clinical practice and can achieve a good eradication effect. However, there are many types of drugs for bismuth-containing quadruple therapy, and patients have greater concerns about the occurrence of adverse drug events which greatly affects medication compliance.

*H. pylori* is an infectious disease; theoretically, sensitive antibiotics should be selected for eradication therapy based on susceptibility testing or the local antibiotic resistance spectrum, which can avoid the abuse of antibiotics, reduce the types of drugs taken, and improve compliance [25,26]. However, the resistance rate of *H. pylori* to amoxicillin is still low, and high-dose dual therapy including PPI combined with amoxicillin has become an increasing concern in recent years. In a clinical study of dual therapy based on systematic review and meta-analysis, 12 randomized controlled studies were included, and the results showed that dual therapy with high-dose PPIs combined with amoxicillin, whether used for primary *H. pylori* eradication or salvage *H. pylori* eradication, could achieve better efficacy, with eradication rates of more than 90%, compared with various traditional radical therapies recommended by current mainstream clinical guidelines [27]. However, whether the above radical treatment options can achieve good results in rural areas of China has not been yet reported.

In this study, for the first time, we conducted a clinical study to determine whether high-dose dual therapy and resistance gene-based triple therapy were superior to empirical bismuth quadruple therapy for *H. pylori* eradication. The results showed that high-dose dual therapy and resistance gene-based triple therapy could achieve an eradication rate equivalent to empirical bismuth quadruple therapy. Due to the reduction in the types of drugs taken and precise radical cures, the adverse events of high-dose dual therapy were much lower than that of empirical bismuth quadruple therapy, and the adverse events of resistance gene-based triple therapy were equivalent to that of empirical bismuth quadruple therapy.

Actually, Swedish scholar Unge et al. [28] first used a PPI and amoxicillin dual regimen to eradicate *H. pylori* as early as 1989, and since then many scholars have attempted different doses and frequencies of PPI and amoxicillin, but consistent and satisfactory eradication rates have remained difficult to obtain. With the deepening of the study on the kinetics and pharmacodynamics of amoxicillin and PPI, the mechanism of the dual regimen has been gradually elucidated. First, *H. pylori* strains are not susceptible to amoxicillin resistance because simultaneous mutations in multiple penicillin-binding protein-related genes cause marked resistance to amoxicillin [29]. Second, amoxicillin is a time-dependent antibiotic. The bactericidal effect mainly depends on the percentage of time when the plasma concentration is higher than the minimum inhibitory concentration. The same amoxicillin dose prolonged the percentage of time when divided into multiple doses. Multiple doses can also increase the local drug concentration in the gastric juice. Oral administration of four times per day can result in a better bactericidal effect [30,31]. Third, amoxicillin exerts a bactericidal effect by inhibiting the formation of bacterial cell walls and is effective only when bacteria multiply actively. *H. pylori* multiplies actively when the intragastric pH is >6.0; thus, amoxicillin has the best bactericidal effect at this pH [5]. In addition, amoxicillin is easily destroyed in an acidic environment, and adequate inhibition of gastric acid is the key to its effective sterilization. As potent antacids, most PPIs are metabolized by CYP2C19 enzymes. When CYP2C19 is a moderate or poor metabolizer, PPI has a better acid-inhibitory effect. When the CYP2C19 gene is an extensive metabolizer, PPI is rapidly metabolized by the body, with a decreased acid-inhibitory effect and decreased *H. pylori* eradication efficacy. Oral PPI can achieve satisfactory acid suppression in CYP2C19 fast, moderate, and poor metabolizers four times daily [30]. The PPI selected for this study was rabeprazole, which is mainly metabolized by non-enzymatic pathways and is less affected by CYP2C19, which can achieve effective acid suppression.

Antibiotic resistance is one of the most important reasons for the failure of *H. pylori* eradication. Our previous epidemiological survey found that the resistance rates to clarithromycin, metronidazole, and levofloxacin in the areas of Yangzhou were 41.0, 38.8, and 44.9%, respectively. The rapid increase in drug resistance may be related to the characteristics of antibiotics that can easily induce drug resistance and their widespread irrational use in mainland China. Therefore, it is essential to understand antibiotic resistance in patients before eradication therapy. This study collected complete antibiotic resistance information for each patient, which was very helpful for us to comprehensively and accurately assess the causes and risk factors of radical cure failure. Our univariate and multivariate analyses of the factors affecting *H. pylori* eradication failure revealed that antibiotic resistance is one of the most important risk factors. In the resistance gene-based triple therapy, we avoided the use of resistant antibiotics and chose sensitive antibiotics to eradicate *H. pylori*. The results showed that a high eradication rate was achieved despite the use of triple therapy, which indicates that it is necessary to select sensitive antibiotics to eradicate *H. pylori* based on resistance results in the areas of China with a high resistance rate. This not only achieves a good eradication effect but also avoids secondary resistance due to antibiotic abuse in rural areas.

We found that most patients were able to alleviate their gastrointestinal discomfort after *H. pylori* eradication. First, almost all patients with *H. pylori* infection have chronic active gastritis, with high acidity in the stomach and a high inflammatory reaction. PPI can increase the pH of the stomach and reverse damage to the gastric mucosa, thus relieving gastric discomfort [32]. Second, microbiota dysbiosis can also cause gastrointestinal discomfort. In a study on the effect of *H. pylori* infection on the gastrointestinal microbiota [33], it was found that the microbial diversity of *H. pylori*-infected patients was significantly lower than that of *H. pylori*-uninfected patients, leading to a disruption of the flora, while successful eradication of *H. pylori* resulted in a transient decrease in microbial diversity due to the use of antibiotics. However, in the long term it significantly increased the microbiota of *H. pylori*-positive patients. The hypogastric microbial richness and homogeneity of the intragastric environment in *H. pylori*-positive patients were similar to those in *H. pylori*-negative patients.

Two notable points were observed in this study. First, we collected comprehensive and complete pre-radical baseline data, including demographic data and clinical characteristics, as well as information on antibiotic resistance in all patients, which increased the reliability of our findings. Second, we chose to collect gastric mucosal samples for molecular biological detection of *H. pylori* virulence genes and drug resistance genes, avoiding the drawbacks of low positive rates and long durations of drug sensitivity test cultures, which cause an inability to routinely perform detection in clinical practice. This method greatly shortened the time of antibiotic resistance results, allowed patients to begin treatment as early as possible, and enabled more extensive individualized diagnosis and treatment in clinical practice.

This study had some limitations. First, it was a prospective, single-center study with a small sample size. Therefore, a multicenter study with a lager sample size is needed to verify the effectiveness of eradication efficacy. Second, we did not detect genetic polymorphisms of CYP2C19 enzyme in patient blood samples. Although we selected rabeprazole, which is less affected by CYP2C19 enzymes, recent studies have shown that rabeprazole metabolism is also affected to some extent by the CYP2C19 genotype, which may have an impact on patients who choose high-dose dual therapy. At present, a potassium-competitive acid blocker (P-CAB) has been selected for *H. pylori* eradication. It is a novel antacid with reversible and competitive inhibitory effects on H^+^, K^+^ ATPase-mediated gastric acid secretion. It is considered to have an antacid effect on PPI and has potential benefits for *H. pylori* eradication. A meta-analysis of 1,599 *H. pylori*-positive patients showed no significant difference in eradication rates between P-CAB-based therapy and conventional PPI therapy in clarithromycin-sensitive patients. However, P-CAB-based therapy was more effective than PPI-based regimens in clarithromycin-resistant patients [34]. Therefore, P-CAB is expected to be a strong first-line therapy for *H. pylori* eradication in the future.

## 5 Conclusions

In summary, in patients with the first-time eradication of *H. pylori* infection in the areas of Yangzhou, high-dose dual therapy and resistance gene-based triple therapy achieved eradication rates and compliance equivalent to those of empirical bismuth quadruple therapy. Meanwhile high-dose dual therapy had lower rates of adverse events, fewer side effects, and greater safety. Eradication of *H. pylori* improves gastrointestinal discomfort in most patients with the infection. Eradication failure is primarily related to antibiotic resistance and poor compliance. Therefore, the appropriate regimen can be individualized for eradication therapy in clinical practice according to the patient’s resistance and tolerance to the drug.
